# *Naegleria fowleri:* Swimming with Death as the Major Outbreak in Pakistan

**Published:** 2019-09

**Authors:** Muhammad NAVEED, Zeshan ALI, Masooma KHWAR, Wahab NAZIR, Nauman KHALID

**Affiliations:** 1. Department of Biotechnology, Faculty of Life Sciences, University of Central Punjab, Lahore, Pakistan; 2. Department of Biotechnology, University of Gujrat Sialkot, Punjab, Pakistan; 3. School of Food and Agricultural Sciences, University of Management and Technology, Lahore, Pakistan

## Dear Editor-in-Chief

*Naegleria fowleri* is commonly known as “brain-eating ameba” is an ameba usually present in swimming pools, rivers, lakes; and causes Primary Amebic Meningoencephalitis (PAM) a lethal brain infection in humans. This infectious agent is found all over the world and some of the deaths are recently reported in Sindh Province of Pakistan. In 2008, first case of *N. fowleri* was reported in Pakistan. Deaths were also reported in 2013–2016. In last four years, 42 deaths have been reported in Pakistan. Chlorination is to be done to kill the organism, while there are no proper chlorination procedures followed in Sindh, Pakistan where most of the cases are reported (1–3).

Infection starts when water containing *N. fowleri* enters the nose and the ameba reaches to the brain along the olfactory nerve. Symptoms start 1–9 d after exposure and causes die 1–18 d after symptoms begin. PAM is difficult to detect because of its rapid progress and usually diagnosis is made after death following hematoxylin and eosin staining of brain. Signs and symptoms of the infection include frontal headache, fever, nausea, vomiting, altered mental status, hallucinations, coma and sometimes-stuffy nose which become fatal within 3–7 d with very few survivors (4).

In Pakistan on Sep 6, 2014, death toll reaches 10 because of primary amebic meningoencephalitis (PAM). Till 2014, at least 14 people lost their lives to complications linked to the amoeba. In 2015, 12 more fatal primary amebic meningoencephalitis cases were reported in the Sindh Province. On Jul 1, 2016, Pakistan reports first *N. fowleri* death in Karachi. Until Aug 18, 2017, Sind’s fifth, death of 2017 reported in Karachi (5).

Health officials tested water of different areas of Karachi which concluded only half of the city is provided with chlorinated water while 90% of pumping houses undergo no chlorination (6). Chlorinated is the only trusted process to kill this organism, proper sanitation should be practiced. Water chlorination level of city was still below than 0.5 ppm, the standard chlorination level recommended by WHO. Deteriorating water line conditions of Karachi hinder in chlorination of water.

No fruitful strategy has yet been defined by Government to combat this deadly pathogen. However, we can keep ourselves safe from this deadly pathogen all we need is to clean our water tanks on regular basis by using disinfectants and bleaches. Avoid swimming in fresh waters reservoirs and unchlorinated swimming pools and we should aware others to follow proper sanitation procedures (3).
